# Student Satisfaction with Audio-Visual Flipped Classroom Learning: A Mixed-Methods Study

**DOI:** 10.3390/ijerph19031053

**Published:** 2022-01-18

**Authors:** Yueh-Chen Yeh

**Affiliations:** Department of Nursing, National Taichung University of Science and Technology, Taichung 40343, Taiwan; yehyc@nutc.edu.tw; Tel.: +886-42219-6957

**Keywords:** flipped classroom, grey relational analysis, learning satisfaction, mixed method

## Abstract

The purpose of this mixed-methods study was to investigate the influential factors of student satisfaction with online digital audio-visual flipped classroom learning. A total of 103 students enrolling in the two-credit compulsory code course participated in this flipped classroom research. Descriptive data analysis and grey relational analysis demonstrated that student respondents were most satisfied with repeated practice (1st), followed immediately by peer learning (2nd), and active learning (3rd). In terms of qualitative data, three themes emerged from the focus group analysis, including: improving independent learning, enhancing peer learning, and increasing teacher–student interaction. The flipped classroom model provided opportunities for students to strengthen their self-directed learning capabilities, improved students’ learning motivation, and to be a team player among third-year nursing students. The results were consistent with the actual circumstance. The results integrated descriptive data, the mathematic model, and interviews to validate the accuracy and rationality of the data. According to this study, an online digital audio-visual flipped classroom could improve student independent learning and enhance peer communication. The results provide an accurate assessment tool suitable for Taiwanese nursing students’ flipped classroom model learning experience.

## 1. Introduction

Flipped classroom models have been popular in the education of health professionals for decades. This model has been widely used in medical and nursing courses, and the flipped approach has been found to positively influence medical and nursing students’ competence in various skills [1]. The flipped classroom model entails redefining the teaching and learning models among educators and students. It aims to maintain the flexibility of the teaching space and makes use of novel strategies for deploying technology and structuring interactions [2]. A flipped classroom can improve students’ learning outcomes, self-directed learning, as well as their knowledge, skills, and satisfaction [1,3,4]. 

Research has demonstrated that a flipped classroom enables students to formulate active learning attitudes, develop nursing skills, and provide simulated clinical scenarios for training medical/nursing students in a safe setting to cultivate their theoretical knowledge and improve the quality of patient care [5,6]. Audio-visual learning has also been found to be pedagogically sound as it encourages a student-centred approach and self-paced learning across disciplines [7]. To implement a flipped classroom, teachers must have both superior teaching concepts with design capabilities, and good technological knowledge and application skills. Audio-visual, supplementary materials often motivate students to engage in learning [8]. In the online digital audio-visual flipped classroom model, content distribution is typically performed via audio-visual prepared by the instructor or through screen capture software such as EverCam (Irish company Camba. tv Ltd, Dublin, Ireland) or HyperCam (Solveig Multimedia, Tomsk, Russian). This allows instructors to make their own digital materials and upload them to digital learning platforms, such as YouTube and other cloud-based platforms, for students to access [9,10]. These learning platforms are critical for the implementation of flipped classrooms. In addition, with the popularisation of the Internet and development of relevant software, short video clips can be recorded for teaching, allowing students to learn the content at their own pace prior to classes and repeatedly watch certain parts as and when required. Thus, both teaching and learning have become flexible and autonomous, thereby helping students formulate lifelong learning skills. Recent studies [10,11] have indicated that online digital audio-visual teaching is welcomed by students and can improve their active learning and engagement, thereby increasing their learning efficacy.

Hence, the data generated from the questionnaire surveys were subjected to a grey relational analysis (GRA). The grey system theory has been used successfully in cases where the information received is uncertain or partial [12]. To address this problem, Deng [12,13] developed the grey system theory as a way to deal with vast unascertained information in various fields, including natural science [12], engineering science [13], social science, education [14,15], and health care service quality factors [16]. GRA uses grey correlations to describe the relationships between degree, strength, and order among influential factors [15].

In this study, nine influential factors related to learning perceptions were analysed with GRA and the results were interpreted. The present study revealed the overall rating of this course and the weight of the following nine factors that affect student learning satisfaction: (1) satisfaction with audio-visual aids, (2) whether the materials are useful, (3) improving active learning, (4) whether the theoretical knowledge and nursing techniques developed meet future needs, (5) peer learning, (6) confidence in applying theoretical knowledge and nursing techniques in clinical practice, (7) improving communication and collaboration skills, (8) self-paced learning, and (9) repeated practice.

This study employed the mixed-methods research design, which combines qualitative and quantitative research approaches for the broad purpose of increasing the breadth and depth of understanding students’ perception regarding the audio-visual flipped classroom model. Learning perceptions and evaluations of the learning approaches are influenced by the nature of the learning environment. The study used descriptive data, the mathematic model, and focus group interviews to capture the contextual learning environment and validate the accuracy and rationality of the data.

## 2. Materials and Methods

This study used sequential explanatory mixed methods, which first involved collecting and analysing quantitative data followed by qualitative data in two consecutive phases within one study [17]. This study used questionnaires and focus group interviews to gain a broad purpose of increasing the breadth and depth of understanding students’ perceptions regarding the audio-visual flipped classroom model [17]. This mixed-methods research aimed to explore students’ perceptions and use these perceptions to improve the pedagogical model.

Third-year college nursing students in the maternal nursing laboratory course were selected for this study through convenience sampling. A total of 103 students from two classes, enrolled in the two-credit compulsory code course, participated in this online digital audio-visual flipped classroom research.

### 2.1. Ethical Considerations

The study was approved by the research ethics board at the institution. Participants provided informed consent before participation. The students were informed that they could withdraw from the study at any time. All personal data were kept confidential. The students signed informed consent forms to allow the recording of the interviews, which were conducted after the semester grades were submitted to the administration office to assure them that their participation had nothing to do with their course grade.

This study had two phases.

### 2.2. First Phase: Quantitative Phase

The first phase involved collecting students’ learning satisfaction with the audio-visual flipped classroom model through questionnaires. The questionnaire was distributed using Google Forms and could be answered anonymously. Questionnaires were collected after each class, and each student submitted four questionnaires.

#### 2.2.1. Instruments

The research instrument was a questionnaire conducted by the author. The structured questionnaire was validated by experts, and their advice was sought when devising the instrument. Questionnaire reliability was established using the test–retest method, yielding a reliability coefficient of 0.91. It contained 10 questions that sought to examine student satisfaction with the audio-visual flipped classroom. Students answered questionnaire items by selecting a score from the Likert rating scale from 1 (strongly disagree) to 5 (strongly agree).

#### 2.2.2. Procedure

##### Before-Class Activities

The researcher used spare time to record the video, write the teaching curriculum, replicate clinical scenarios, and then asked students to watch the video prior to the class, at their convenience. The audio-visual content was uploaded to the cloud, five days prior to the class. The pre-recorded video primarily focused on clinical scenarios and nursing skill practice procedures.

##### In-Class Activities

- Students were divided into eight teams and asked to participate in a given scenario. Each session consisted of two phases. During the skill practice session, student groups had 15 min to practice nursing skills and provide care, through the scenario. Simultaneously, the other students could observe the live action through a camera from another room.
- During the second phase or debriefing, verbal feedback and comments were provided for each student team and 15 min were dedicated to answering questions and debriefing. In the assessment phase, which took place the following week, each student was required to take a formal exam and perform the nursing technique and procedure individually under the instructor’s observation.

##### After-Class Activities

At the end of the course, students were required to write a summary of their course learning. This formed the basis for the analysis of “learning effectiveness.” Additionally, students completed the learning satisfaction questionnaire using Google Forms.

### 2.3. Second Phase: Qualitative Phase

The second phase was done with focus group interviews by the author. A research poster was posted in the public area one month before the beginning of the data collection. Students were invited to discussions of learning perceptions to gain a more in-depth understanding of their experience with an audio-visual flipped classroom model.

### 2.4. Data Collection

An interview guide was prepared to understand the learning experience from students’ perspectives and open-ended questions were asked to provide in-depth information and clarify the possibility of misinterpretation. The study was guided by the following questions:

1. Did you watch the video clips?
2. (If students had not watched the video) Why did you not watch the video?
3. (If students had watched the video) How many times did you watch the video clips, and how much time did you spend on them?
4. Did you find the video content difficult to comprehend? Was the content clear?
5. What do you think of the audio-visual flipped classroom teaching method?

To ensure that interviewees had enough time to express their thoughts and experiences, the interviews were allotted 45–60 min.

### 2.5. Data Analysis

The questionnaire answers were first computed for the mean, standard deviation (SD), and median (interquartile range). The statistical package for SPSS 26.0 (IBM Corp., Armonk, NY, USA) was used. A total of 103 students completed the questionnaire surveys. However, the student respondents may not have been normally distributed; this may have led to interpretation inadequacies. Grey Model (GM) (0, N), a special type of multiple regression modelling that is distinct from traditional models, was applied to represent the dynamic data sequence, which can help supplement what the descriptive statistics fail to reveal [15,16]. GM (0, N) model of the grey system, a soft computing, was used to examine the individual weight of the nine affecting factors of student learning satisfaction in a flipped classroom. Therefore, both descriptive data analysis and GM (0, N) of the grey system were employed for analysing and comparing factor weight

In terms of qualitative data analysis, this study employed Creswell’s multistep analysis technique [17]. Manual analysis was used to read the interview transcripts repeatedly to process code phrases, label categories, and generate themes. First, the focus group interview notes were transcribed by listening to the original recording. Prior to coding, the author read the participants’ transcripts line by line several times and identified the key messages of each line to develop a comprehensive understanding of their responses. Subsequently, the data were repeatedly reviewed and coded by the author. Next, codes of the same nature were linked to form a higher-level concept, that is, a category, which was then bundled into a theme. These steps facilitated a rigorous analytical process to develop and enrich data interpretation [17].

### 2.6. Rigor

The author conducted and transcribed the interviews and analysed the data. Once the data analysis was completed, it was verified by inviting two interviewees to conduct member checking. The inspection of the text information was carried out repeatedly by the author to increase the depth and breadth of the theme. A peer review was also performed by asking an Assistant Professor, who is qualified in qualitative research, to review the data analysis process. The author and the Assistant Professor independently analysed the data, compared the categories and themes, and resolved the discrepancies. To ensure the rigour of the mixed-methods analysis, the data integration involved building and merging integration techniques, and the data were analysed separately and independently [18,19]. The internal consistency and reliability of the questionnaire was ensured with expert consultations. The use of established data analysis processes provides an audit trail, which ensures trustworthiness and credibility [18].

## 3. Results

A total of 103 students (97 women and 6 men) from two classes completed the anonymous learning satisfaction surveys. The participants were aged between 18 and 19 years. Four cycles of the audio-visual flipped classroom model were conducted with the participants in the class between September 2020 and January 2021 during the maternal nursing laboratory course.

### 3.1. Quantitative Results

The questionnaire aimed to investigate factors affecting students’ learning satisfaction with audio-visual flipped classroom learning. Data analysis revealed that the audio-visual flipped classroom learning model has multiple advantages.

#### 3.1.1. Descriptive Data

**Figure 1:**
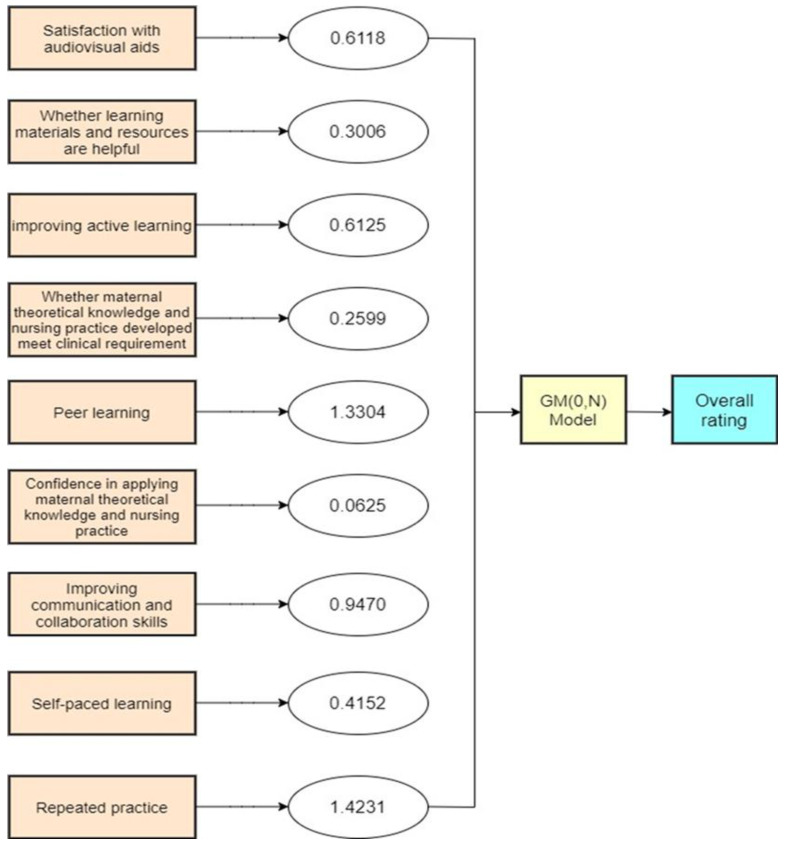
Grey Model (GM) (0, N) the significance of nine factors.

Learning satisfaction mean scores increased over time. The top three ratings for learning satisfaction were “repeated practice” (4.28; SD = 0.16), “peer learning” (4.25; SD = 0.45), and “active learning” (4.18; SD = 0.22) (Table 1). The significance of nine influential factors was calculated by GRA (Figure 1).

#### 3.1.2. GM (0, N) Model for Relational Analysis: Formatting of Mathematical Components

##### GM (0, N) Model for Relational Analysis

The main function of the GM (0, N) model, a specific instance of the GM (1, N) model, aims at analysing the quantitative relationship among N variables. As a static factor analysis, it undergoes the following procedure:

(1) $$\alpha z_{1}^{\left(1\right)}\left(k\right)=\sum_{j=2}^{N}bjx_{j}^{\left(1\right)}\left(k\right)=b_{2}x_{2}^{\left(1\right)}\left(k\right)+b_{3}x_{3}^{\left(1\right)}\left(k\right)+\ldots +b_{N}x_{N}^{\left(1\right)}\left(k\right)$$

with:

$$z_{1}^{\left(1\right)}\left(k\right)=0.5x_{1}^{\left(1\right)}\left(k-1\right)+0.5x_{1}^{\left(1\right)}\left(k\right),\;k=2,\;3,\;4,\;\ldots ,\;n$$

1. Substitute each value, we can obtain

(2) $$\alpha_{1}z_{1}^{\left(1\right)}\left(2\right)=b_{2}x_{2}^{\left(1\right)}\left(2\right)+\ldots +b_{N}x_{N}^{\left(1\right)}\left(2\right)\;\alpha_{1}z_{1}^{\left(1\right)}\left(3\right)=b_{2}x_{2}^{\left(1\right)}\left(3\right)+\ldots +b_{N}x_{N}^{\left(1\right)}\left(3\right)\;\ldots \ldots \ldots \ldots \ldots \ldots \ldots \ldots \ldots \;\alpha_{1}z_{1}^{\left(1\right)}\left(n\right)=b_{2}x_{2}^{\left(1\right)}\left(n\right)+\ldots +b_{N}x_{N}^{\left(1\right)}\left(n\right)$$

2. Divide both sides of the equation by $\alpha_{1}$, then convert it into a matrix form

(3) $$\left[\begin{array}{c} 0.5x_{1}^{\left(1\right)}\left(1\right)+0.5x_{1}^{\left(1\right)}\left(2\right)\\ 0.5x_{1}^{\left(1\right)}\left(2\right)+0.5x_{1}^{\left(1\right)}\left(3\right)\\ \begin{array}{c} \vdots \\ 0.5x_{1}^{\left(1\right)}\left(n-1\right)+0.5x_{1}^{\left(1\right)}\left(n\right) \end{array} \end{array}\right]=\left[\begin{array}{c} x_{2}^{\left(1\right)}\left(2\right)\;\cdots \;x_{n}^{\left(1\right)}\left(2\right)\\ x_{2}^{\left(1\right)}\left(3\right)\;\cdots \;x_{n}^{\left(1\right)}\left(3\right)\\ \begin{array}{c} \vdots \;\;\;\;\;\;\;\;\;\;\;\cdots \;\;\;\;\;\;\;\;\;\;\;\vdots \\ x_{2}^{\left(1\right)}\left(n\right)\;\cdots \;x_{n}^{\left(1\right)}\left(n\right) \end{array} \end{array}\right]\left[\begin{array}{c} \frac{b_{2}}{\alpha_{1}}\\ \begin{array}{c} \frac{b_{3}}{\alpha_{1}}\\ \frac{b_{4}}{\alpha_{1}}\\ \begin{array}{c} \vdots \\ \frac{b_{N}}{\alpha_{1}} \end{array} \end{array} \end{array}\right]$$

If $\frac{b_{j}}{a_{1}}$ = ${\hat{b}}_{m}$ with m = 2, 3, 4, …, *N*, then (3) becomes

(4) $$\left[\begin{array}{c} 0.5x_{1}^{\left(1\right)}\left(1\right)+0.5x_{1}^{\left(1\right)}\left(2\right)\\ 0.5x_{1}^{\left(1\right)}\left(2\right)+0.5x_{1}^{\left(1\right)}\left(3\right)\\ \begin{array}{c} \vdots \\ 0.5x_{1}^{\left(1\right)}\left(n-1\right)+0.5x_{1}^{\left(1\right)}\left(n\right) \end{array} \end{array}\right]=\left[\begin{array}{c} x_{2}^{\left(1\right)}\left(2\right)\;\cdots \;x_{n}^{\left(1\right)}\left(2\right)\\ x_{2}^{\left(1\right)}\left(3\right)\;\cdots \;x_{n}^{\left(1\right)}\left(3\right)\\ \begin{array}{c} \vdots \;\;\;\;\;\;\;\;\;\;\;\cdots \;\;\;\;\;\;\;\;\;\;\;\vdots \\ x_{2}^{\left(1\right)}\left(n\right)\;\cdots \;x_{n}^{\left(1\right)}\left(n\right) \end{array} \end{array}\right]\left[\begin{array}{c} {\hat{b}}_{2}\\ \begin{array}{c} {\hat{b}}_{3}\\ {\hat{b}}_{4}\\ \begin{array}{c} \vdots \\ {\hat{b}}_{N} \end{array} \end{array} \end{array}\right]$$

Likewise, use a matrix solution to solve the values of ${\hat{b}}_{m}$ (with) $\hat{B}$ = ($Y^{T}Y{)}^{-1}Y^{T}X$

(5) $$X=\left[\begin{array}{c} 0.5x_{1}^{\left(1\right)}\left(1\right)+0.5x_{1}^{\left(1\right)}\left(2\right)\\ 0.5x_{1}^{\left(1\right)}\left(2\right)+0.5x_{1}^{\left(1\right)}\left(3\right)\\ \begin{array}{c} \vdots \\ 0.5x_{1}^{\left(1\right)}\left(n-1\right)+0.5x_{1}^{\left(1\right)}\left(n\right) \end{array} \end{array}\right],Y=\left[\begin{array}{c} x_{2}^{\left(1\right)}\left(2\right)\;\cdots \;x_{n}^{\left(1\right)}\left(2\right)\\ x_{2}^{\left(1\right)}\left(3\right)\;\cdots \;x_{n}^{\left(1\right)}\left(3\right)\\ \begin{array}{c} \vdots \;\;\;\;\;\;\;\;\;\;\;\cdots \;\;\;\;\;\;\;\;\;\;\;\vdots \\ x_{2}^{\left(1\right)}\left(n\right)\;\cdots \;x_{n}^{\left(1\right)}\left(n\right) \end{array} \end{array}\right],\hat{B}=\left[\begin{array}{c} {\hat{b}}_{2}\\ \begin{array}{c} {\hat{b}}_{3}\\ {\hat{b}}_{4}\\ \begin{array}{c} \vdots \\ {\hat{b}}_{N} \end{array} \end{array} \end{array}\right]$$

The value of ${\hat{b}}_{m}$ refers to the weight value of compare sequence to standard sequence $x_{1}$

Calculation Procedure

(1) Create the original sequence

$$x_{1}^{\left(0\right)}=J=(4,\;4,\;4,\ldots \;,\;5,\;5,\;5)$$

$$x_{2}^{\left(0\right)}=A=(5,\;4,\;4,\ldots ,\;5,\;5,\;4)$$

$$x_{3}^{\left(0\right)}=B=(4,\;4,\;4,\ldots \;,\;5,\;5,\;5)$$

$$x_{4}^{\left(0\right)}=C=(5,\;4,\;4,\ldots \;,\;5,\;5,\;5)$$

$$x_{5}^{\left(0\right)}=D=(5,\;4,\;4,\ldots \;,\;5,\;5,\;4)$$

$$x_{6}^{\left(0\right)}=E=(5,\;4,\;5,\ldots \;,\;5,\;5,\;5)$$

$$x_{7}^{\left(0\right)}=F=(5,\;4,\;5,\ldots \;,\;5,\;5,\;5)$$

$$x_{8}^{\left(0\right)}=G=(5,\;4,\;5,\ldots \;,\;5,\;5,\;5)$$

$$x_{9}^{\left(0\right)}=H=(4,\;4,\;5,\;\ldots ,\;5,\;5,\;5)$$

$$x_{10}^{\left(0\right)}=I=(4,\;4,\;4,\ldots \;,\;5,\;5,\;5)$$

(2) Create Accumulated Generating Operation (AGO) original sequence

$$\begin{array}{c} x_{1}^{\left(1\right)}=J=(4,\;8,\;12,\;17,\;\ldots ,\;453,\;458,\;463)\\ z_{1}^{\left(1\right)}=(-------,\;6,\;10,\;14.5,\;19.5,\;\ldots ,\;450.5,\;455.5,\;460.5)\\ x_{2}^{\left(1\right)}=A=(5,\;9,\;13,\;17,\;\ldots ,\;433,\;438,\;442)\\ x_{3}^{\left(1\right)}=B=(4,\;8,\;12,\;16,\ldots \;,\;427,\;432,\;437)\\ x_{4}^{\left(1\right)}=C=(5,\;9,\;13,\;17,\ldots \;,\;421,\;426,\;431)\\ x_{5}^{\left(1\right)}=D=(5,\;9,\;13,\;17,\ldots \;,\;431,\;436,\;440)\\ x_{6}^{\left(1\right)}=E=(5,\;9,\;14,\;18,\ldots \;,\;435,\;440,\;445)\\ x_{7}^{\left(1\right)}=F=(5,\;9,\;14,\;18,\ldots \;,\;446,\;451,\;456)\\ x_{8}^{\left(1\right)}=G=(5,\;9,\;14,\;18,\ldots \;,\;429,\;434,\;439)\\ x_{9}^{\left(1\right)}=H=(4,\;8,\;13,\;17,\;\ldots ,\;423,\;428,\;433)\\ x_{10}^{\left(1\right)}=I=(4,\;8,\;12,\;16,\ldots \;,\;437,\;442,\;447) \end{array}$$

(3) Substitute the above values into the equation of GM (0, N)

$$\left[\begin{array}{c} 10\\ 14.5\\ 19.5\\ \vdots \\ 450.5\\ 455.5\\ 460.5 \end{array}\right]\left[\begin{array}{ccccc} 9 & 8 & \cdots & 8 & 8\\ 13 & 12 & \cdots & 13 & 12\\ 17 & 16 & \cdots & 17 & 16\\ \vdots & \vdots & \ddots & \vdots & \vdots \\ 433 & 427 & \cdots & 423 & 437\\ 438 & 432 & \cdots & 428 & 442\\ 442 & 437 & \cdots & 433 & 447 \end{array}\right]\left[\begin{array}{c} {\hat{b}}_{2}\\ {\hat{b}}_{3}\\ {\hat{b}}_{4}\\ \vdots \\ {\hat{b}}_{7}\\ {\hat{b}}_{8}\\ {\hat{b}}_{9} \end{array}\right]$$

Using a matrix solution to solve the values of ${\hat{b}}_{m}$ (with) $\hat{B}$ = ($Y^{T}Y{)}^{-1}Y^{T}X$,

$$X=[\begin{array}{c} 10\\ 14.5\\ 19.5\\ \vdots \\ 450.5\\ 455.5\\ 460.5 \end{array}]Y=[\begin{array}{ccccc} 9 & 8 & \cdots & 8 & 8\\ 13 & 12 & \cdots & 13 & 12\\ 17 & 16 & \cdots & 17 & 16\\ \vdots & \vdots & \ddots & \vdots & \vdots \\ 433 & 427 & \cdots & 423 & 437\\ 438 & 432 & \cdots & 428 & 442\\ 442 & 437 & \cdots & 433 & 447 \end{array}],\hat{B}=[\begin{array}{c} {\hat{b}}_{2}\\ {\hat{b}}_{3}\\ {\hat{b}}_{4}\\ \vdots \\ {\hat{b}}_{7}\\ {\hat{b}}_{8}\\ {\hat{b}}_{9} \end{array}]$$

Figure 1 shows the significance of nine factors from Grey Model (0, N) weight analysis.

### 3.2. Qualitative Results

From the two classes, 48 students (45 women and 3 men) voluntarily participated in the focus groups; a total of 17, 12, and 19 students participated in the three focus groups, respectively. Three focus groups were conducted between January 2021 and February 2021 after the semester grades were submitted to the administration office.

Three main themes were identified from the three focus groups: improving independent learning, enhancing peer learning, and increasing teacher–student interaction. The transcripts anonymised the student quotations. Each of these themes had subthemes. The themes highlighted and supported our questionnaire survey findings and provided insight into the use of an audio-visual flipped classroom model.

#### 3.2.1. Improving Independent Learning

During the learning process, students often need to realign their thoughts, feelings, and motivations to apply the acquired knowledge in clinical practice. In this study, the participants found the online audio-visual course helpful in improving their independent learning.

*In traditional laboratory courses, teachers would demonstrate nursing skills in one class and then ask us [students] to repeat the procedure in the next class. Honestly, this did not give us enough time to absorb the skill content and remember the procedure; Traditionally, only one teacher would be running the laboratory class and would be too busy to take care of all students during the class. By contrast, the audio-visual flipped classroom allows for rewinding the video and repeatedly watching certain part any time. I can learn at home by myself. (No. 2.01)*

##### Developing a Prep Habit

Self-directed learning is an important aspect of flipped classrooms. Students are required to read the content by themselves prior to the class; this is intended to prepare them to gain new knowledge with enthusiasm. When teaching activities previously conducted in the classroom are replaced with flipped classroom activities, now conducted beforehand, a passive learning culture is supplanted by an active “learner-centred” learning culture. Consequently, students understand their learning needs and their learning efficacy, and their sense of achievement is enhanced.

*Watching the video before the class helped me understand the content of the upcoming laboratory class. In addition, watching a video of the content that will be taught in the class-room beforehand, helps me memorise it better, and parts that are unclear in the video can be clarified during class. (No. 2.03)*

Students who did not watch the audio-visual clips in advance, still believed that these video clips were helpful for self-learning.

*Honestly, I did not watch the videos before each class. However, before taking formal exams, I would watch the online videos, as they can help clarify some of my questions and confusion. In addition, knowing that there are videos for me to watch at home, reduces any sense of panic (before taking the exam). (No. 1.11)*

*To practice each nursing skill, I watched the videos at least three times. Furthermore, I watched the video repeatedly before the class or practical exams. (No. 1.03)*

##### Creating a Flexible Learning Environment

Notwithstanding individual variations, such as different learning motivations and concentration levels during class, students generally agreed that to keep up with the flipped classroom, they had to preview the teaching material and learn independently. In addition, students also concurred that the flipped classroom provided them with flexible learning opportunities, allowing them self-learning anytime and anywhere.

*With videos uploaded to the website, I can access them and practice anytime and anywhere. For example, I would watch a video in bed for 20 min after taking a shower at night and then practice once I memorised it. I think [video teaching] is convenient, as you can watch it in bed, which motivates me to learn. (No. 2.04)*

By creating a “flexible learning environment”, students are not restricted by their location and, therefore, can adapt to self-directed learning. If teachers record their teachings as short video clips that can be made available prior to the class, audio-visual content may also assist students to memorise clinical practice or procedural skills. In addition, students have the flexibility to learn at their own pace and watch the videos as often as they wish.

#### 3.2.2. Enhancing Peer Learning

Most students mentioned that the audio-visual flipped classroom made learning more pleasant, valuable, and practical, but they also felt tense in some aspects. It also allowed them to practice nursing skills, crucial to patient care, in risk-free clinical scenarios.

*For skills that must be practiced, the teacher not only simulated the clinical scenario but also marked points that required attention. Consequently, when I watched the video, I knew what the key points and critical operations of the skill were. (No. 1.05)*

*All the time, we [students] discussed how to practice nursing skills and take care of patients. It’s challenging and kind of helpful. (No. 2.07)*

*Learning from other classmates facilitated the learning process. (No. 1.06) During group practice, I have been thinking that, if I had to perform the procedure of nursing technique, would I be doing it the same way? (No. 2.09)*

*While watching teammates perform the assigned situation, I realised that I was not the only one who did not know how to take care of a particular scenario. That made me feel relaxed. (No. 1.05)*

##### Increasing the Opportunity for Group Discussions

The majority of students appreciated the skills practiced in group simulations. Learning through doing and observing others is an advantage, particularly in a laboratory class. Despite feeling tense during group discussions and debriefing sessions, students felt supported by their team members. This proved to be an added opportunity to strengthen communication among team members and it reinforced the flipped classroom learning.

Regarding benefits of peer learning:

*Working with a partner helped me learn about students who are smarter than me and learn how to work quickly and efficiently. (No. 1.01)*

Group discussions helped students’ personal growth by increasing peer interactions and facilitating the development of communication and cooperation skills. Additionally, peer learning improved their nursing skills; this is crucial because the core of nursing care is teamwork and multidisciplinary collaboration. Furthermore, skills could be integrated into the clinical context through clinical practice and the flipped classroom learning experience enabled students to identify how they would respond in simulated conditions of patient care.

#### 3.2.3. Increasing Teacher–Student Interaction

The flipped classroom improved both discussions between students and teachers, and interactions among the students. Increased opportunities for teachers to engage with students enhanced the teacher–student relationship.

*From the flipped classroom designed by my teacher, I could feel the teacher’s dedication and intention to constantly teach us new things. At least in our department, my teacher was the first to implement a flipped classroom the teaching was great. (No. 2.05)*

##### Feeling the Teacher’s Passion for Teaching

The author, as a professional educator, reflected on his/her own teaching attitudes and methods of responding to ever-changing trends and student needs. The author, as an instructor, received increased respect from the students for professionalism, pursuit of knowledge, and appreciation for employing effective teaching methods. These outcomes are particularly important in nursing education as the aim is to train students to become skilled and caring nurses.

*I appreciated the teacher’s effort in helping us with the new model. The teacher was very lively during classes and seemed very close to us. Plus, her teaching was clear, concise, and complete, making the class interesting and stress-free. (No. 1.11)*

By listing to learning goals and critical behaviours in the video, students could clearly understand the key points of the content as well as skills to be evaluated, thereby reducing their exam related stress.

*Compared with other subjects, I can, now, better understand the steps involved in clinical maternity nursing. Since the teacher always incorporated many clinical events into the class and practical sessions, learning and practicing maternal care was less boring. This helped connect the material to the clinical scenario. (No. 2.13)*

##### Making Learning Faster

The flipped classroom promoted learning autonomy among the students and converted them from passive spectators to active participants through group exercises, exams, activities, peer evaluation, and reflection. This transformed the classroom into a superior learning environment for both teachers and students.

*The teacher would discuss problems immediately to help us understand the issues that we had neglected. Consequently, we are likely to better remember these issues in formal exams or internships in the future and avoid making the same mistakes. (No. 1.09)*

This teaching strategy enables the use of mixed methods in teaching. The videos or the written content can be applied to convey theoretical knowledge while physical courses can be used to strengthen theoretical knowledge through individual discussions, problem solving, and collaborative skill practice. The flipped classroom restores learning autonomy to students and reinforces active participation, thereby creating a superior learning environment for both teachers and students.

### 3.3. Data Integration

The integrated data are discussed in this section. The qualitative results were generally supported by the data generated from the quantitative findings; for example, the flipped classroom received positive feedback from the students. The quantitative results indicated that most students in the flipped classroom had relatively high levels of learning satisfaction. The top three ratings for the learning satisfaction scores were ‘repeated practice’, ‘peer learning’, and ‘improving active learning’. This study conducted focus group interviews to understand students’ learning perceptions of audio-visual flipped classrooms. Table 2 presents this joint display [17] and provides insights into how students’ attitudes and satisfaction may impact their learning perceptions.

## 4. Discussions

Improving the quality of education and enhancing the quality of students’ learning are among the main concerns of educators. A rational evaluation of students’ learning perceptions are an important means of maintaining the steady development of the education system. The purpose of this study was to assess whether an audio-visual flipped classroom model could help establish critical thinking and promote self-directed learning among students. Mixed methods are increasingly being used in clinical nursing research and nursing education studies [17,18]. The results from the quantitative data provided a general understanding of students’ learning perspectives and efficiency changes over time. The focus group interviews conducted at the end of the semester enabled an in-depth exploration of the students’ views. This study’s questionnaire and focus group interviews generated different yet complementary data. The relatively high satisfaction, self-directed learning, and survey scores were supported by results from the focus group interviews, which helped the author to interpret the quantitative data related to those outcomes [17]. The flipped classroom is considered a self-directive learning methodology that encourages higher-order thinking and active participation from students. The results of this study can help educators understand students’ perceptions and acceptance of audio-visual flipped classrooms in maternal nursing laboratory courses as well as in other professional nursing courses. However, the evaluation process requires the selection of the appropriate method of evaluation, according to the needs of the evaluation object. This study applied the GRA, which enabled the author to evaluate the ambiguity and uncertainty of factors affecting students’ learning perceptions derived from the Likert-type scale survey, and set different weights for the data [20,21]. Therefore, correlation theory and evaluation results have strong objectivity and rationality [14,15].

The students’ responses indicate that, by asking students to learn the necessary skills and clinical scenarios prior to the class, the audio-visual flipped classroom allowed students to participate in a simulated clinical environment, raise questions, and engage in discussions with the teacher, online or in person, thereby improving their involvement in learning. Most students who engaged with the digital flipped classroom maintained a positive attitude toward this teaching method from both quantitative and qualitative data. It allowed the teachers to utilise precious class time for individual discussions with students. The results of this study are consistent with those of previous studies [21,22], which found that college students accept the student-centred concept of the flipped classroom model and believe it can improve their involvement in learning. In addition to improving students’ learning motivation, a digital flipped classroom provides students with an opportunity to participate in discussions, a skill imperative to providing patients with safe medical environment. Youhasan [1] suggested that the professionalism of nursing teachers is crucial. Teachers must simulate clinical situations where students can learn to apply their knowledge, skills, and attitudes towards patient care. Furthermore, teachers should guide students to reflect on the quality of care they have delivered. These are key to successful implementation of a digital flipped classroom [2].

The actual implementation of a flipped classroom varies for each teacher [5]. As indicated by Bergmann and Sams [2], successful implementation relies on helping students to engage in learning and returning their learning autonomy to them. In the classroom, students should be provided with more opportunities to discuss questions and cultivate critical thinking. This can engage them in adaptive learning at higher levels, thereby maximising learning outcomes. The teaching method of the audio-visual flipped classroom is in accordance with the learning style of modern-day students, and teachers must be ready to adapt to the ever-changing educational environment and trends incorporating technological tools into their teaching design [11].

Rather than mechanically memorising operational steps, students could learn in an immersive manner during practical nursing courses. This is consistent with the idea proposed by researchers [5], mentioning that nursing courses should be “lively and interesting”. By simulating clinical scenarios in digital teaching materials, the flipped classroom connected classes with clinical scenarios, improving the link between students’ practices and lectures, thus, reducing the gap between learning and application [4]. Students reported that the digital flipped classroom provided them with flexible learning opportunities and allowed them to conduct self-study as per their time and convenience. This indicates that students who receive course materials in advance can prepare for upcoming topics. The finding that the flipped classroom was preferred by students over traditional teaching methods is consistent with a previous study [22] that demonstrated that flipped classrooms help students to better understand material and learn more about the course, hence, improving learning performance [23]. During the focus group interview, students mentioned that the audio-visual flipped classroom increased their interest in learning, created a positive learning atmosphere, and established a harmonious teacher–student relationship. Therefore, the delivery of a maternal nursing laboratory course in the flipped classroom model provided students with opportunities to repeatedly practice nursing skills. Consequently, students could familiarise themselves with different clinical scenarios and conduct self-learning in a safe environment [11].

### Limitations

This study makes a valid and strong contribution to the literature by addressing the teaching strategy of using a digital audio-visual flipped classroom model, which helps to improve student independent learning and enhance peer communication. However, this study has some limitations. First, there was no control group, which limits the external validity of the results. Second, the study did not conduct pre-intervention surveys; thus, the results may partly reflect the influence of a new learning/teaching strategy, rather than the influence of the flipped classroom model. It should also be noted that all results related to improving learning and the effectiveness of learning is based on the students’ self-report and not on independent measures, such as quiz scores or academic performance. Third, the researcher is a faculty member, which may have also influenced how students answered the questions in the interview. Future studies on the effects of a flipped classroom should address these limitations and explore the actual performance of students.

## 5. Conclusions

As teaching spaces become more flexible with technology, offering multiple possibilities in student interactions, a flipped classroom has the potential to enhance both interactions between students and teachers, and among students. Technological developments have facilitated the delivery of theoretical knowledge and have allowed us to apply effective teaching strategies. The results of this study provide a reference for the implementation of audio-visual flipped classrooms in practical maternal nursing laboratory courses. This student-centred teaching approach can also be successfully extended to other nursing subjects and healthcare training settings.

## Figures and Tables

**Table 1:** Descriptive data of learning satisfaction (*n* = 103).

	Factor	Mean (SD)	Median (Interquartile Range)
A	Satisfaction with audio-visual aids	4.13 (0.13)	4 (4–5)
B	Whether learning materials and resources are helpful	4.08 (0.19)	4 (3–5)
C	Improving active learning	4.18 (0.56)	4 (3–4)
D	Whether maternal theoretical knowledge and nursing practice developed meet clinical requirement	4.06 (0.13)	4 (3–4)
E	Peer learning	4.25 (0.33)	4 (4–5)
F	Confidence in applying maternal theoretical knowledge and nursing practice	3.91 (0.29)	4 (3–4)
G	Improving communication and collaboration skills	4.04 (0.22)	4 (3–4)
H	Self-paced learning	4.11 (0.28)	4 (4–5)
I	Repeated practice	4.28 (0.16)	4 (4–5)
J	Overall rating	4.25 (0.62)	4 (4–5)

Standard deviation = SD.

**Table 2:** Joint Display of Students’ Learning Perceptions.

Learning Satisfaction	Mean(SD)	Weighting	Students’ Perceptions
Repeated practice	4.28 (0.16)	1.4231	Emphasise satisfaction for repeatedly watching certain parts at any time Helps to learn at any moment Helps to preview the teaching material and learn independently Helpful for self-learning Feel the video content is clear
Self-paced learning	4.11 (0.28)	0.4152	
Satisfaction with audio-visual aids	4.13 (0.13)	0.6118	
Peer learning	4.25 (0.33)	1.3304	Helps with peer-assisted learning Increases the opportunities for group interactions and discussions, although group discussions were stressful Helps with learning actively from each other
Improving communication and collaboration skills	4.04 (0.22)	0.9470	
Improving active learning	4.18 (0.56)	0.6125	Helps to gain knowledge independently at their own pace Helps to improve self-directed learning Helps to integrate into the clinical context Helps to improve the overall course engagement Helpful for future clinical practice
Confidence in applying maternal theoretical knowledge and nursing practice	3.91 (0.29)	0.0625	
Maternal theoretical knowledge and nursing practice developed meet clinical requirement	4.06 (0.13)	0.2599	

## Data Availability

The data presented in this study are available on request from the author. The data are not publicly available due to ethical restrictions.

## References

[1] Youhasan P., Chen Y., Lyndon M., Henning M.A. (2021). Exploring the pedagogical design features of the flipped classroom in undergraduate nursing education: A systematic review. BMC Nurs..

[2] Bergmann J., Sams A. (2012). Flip Your Classroom: Reach Every Student in Every Class Every Day.

[3] McLaughlin J.E., Roth M.T., Glatt D.M., Gharkholonarehe N., Davidson C.A., Griffin L.M., Esserman D.A., Mumper R.J. (2014). The flipped classroom: A course redesign to foster learning and engagement in a health professions school. Acad. Med..

[4] Yeh Y.C. (2016). Undergraduate nursing students’ perceptions of high fidelity simulation-based learning. Int. Arch. Nurs. Health. Care.

[5] Huang H.M., Cheng S.F. (2018). Application of flipped classroom teaching strategy in nursing education. Hu Li Za Zhi.

[6] Ding C., Li S., Chen B. (2019). Effectiveness of flipped classroom combined with team-, case-, lecture-and evidence-based learning on ophthalmology teaching for eight-year program students. BMC Med. Educ..

[7] Malhotra S., Goyal A.K. (2013). Role of online education in modern education system. Int. J. Res. Manag. IT.

[8] Leonard D. (2015). Great Expectations: Students and Video in Higher Education; Sage White Paper. https://studysites.sagepub.com/repository/binaries/pdfs/StudentsandVideo.pdf.

[9] Schwartz T.A. (2014). Flipping the statistics classroom in nursing education. J. Nurs. Educ..

[10] McLean S., Attardi S.M., Faden L., Goldszmidt M. (2016). Flipped classrooms and student learning: Not just surface gains. Adv. Physiol. Educ..

[11] Green R.D., Schlairet M.C. (2017). Moving toward heutagogical learning: Illuminating undergraduate nursing students’ experiences in a flipped classroom. Nurse Educ. Today.

[12] Deng J. (1982). Control problems of grey systems. Syst. Control. Lett..

[13] Deng J. (1989). Introduction to grey system theory. J. Grey Syst..

[14] Chen C.H., Tzeng G.H. (2011). Assessment model for improving educational curriculum materials based on the DANP Technique with Grey Relational Analysis. Int. J. Inf. Syst. Logist. Manag..

[15] Zhang X., Yang X., Yang J. (2021). Teaching Evaluation Algorithm Based on Grey Relational Analysis. Complexity.

[16] Aydemir E., Sahin Y. (2019). Evaluation of healthcare service quality factors using grey relational analysis in a dialysis center. Grey Syst. Theory Appl..

[17] Creswell J.W., Vicki L., Plano C. (2011). Designing and Conducting Mixed Methods Research.

[18] Schoonenboom J., Johnson R.B. (2017). How to construct a mixed methods research design. Kölner Z. Soziol. Soz..

[19] Busebaia T.J.A., John B. (2020). Can flipped classroom enhance class engagement and academic performance among undergraduate pediatric nursing students? A mixed-methods study. Res. Pract. Technol. Enhanc. Learn..

[20] Yamaguchi D., Li G.D., Nagai M. (2007). Verification of Effectiveness for Grey Relational Analysis Models. J. Grey Syst..

[21] Wu C.H. (2007). On the application of grey relational analysis and RIDIT analysis to Likert scale surveys. Int. Math. Forum.

[22] Tang T., Abuhmaid A.M., Olaimat M., Oudat D.M., Aldhaeebi M., Bamanger E. (2020). Efficiency of flipped classroom with online-based teaching under COVID-19. Interact. Learn. Environ..

[23] Missildine K., Fountain R., Summers L., Gosselin K. (2013). Flipping the classroom to improve student performance satisfaction. J. Nurs. Educ..

